# Drug-Resistant Tuberculosis and COVID-19: A Scoping Review on a New Threat to Antimicrobial Resistance

**DOI:** 10.1590/0034-7167-2022-0803

**Published:** 2023-12-04

**Authors:** Beibilene Perlato Melo da Silva, Anelisa Soares de Almeida, Matheus Gabriel de Melo Sérgio, Thamires Carraro Gatto, Vinícius Paglione Carasek, Mellina Yamamura

**Affiliations:** I Universidade Federal de São Carlos. São Carlos, São Paulo, Brazil

**Keywords:** Tuberculosis, Multidrug-Resistant Tuberculosis, Extensively Drug-Resistant Tuberculosis, Microbial Drug Resistance, COVID-19, Tuberculosis, Tuberculosis Resistente a Múltiples Medicamentos, Tuberculosis Extensivamente Resistente a Drogas, Farmacorresistência Microbiana, COVID-19, Tuberculose, Tuberculose Resistente a Múltiplos Medicamentos, Tuberculose Extensivamente Resistente a Medicamentos, Resistência Microbiana a Medicamentos, COVID-19

## Abstract

**Objective:**

To assess the impact of COVID-19 on the morbidity and mortality associated with drug-resistant tuberculosis (DR-TB).

**Methods:**

A comprehensive review of articles published in international databases since December 2019 was conducted. The findings are presented in a narrative format, supplemented with tables, diagrams, and a map created using ArcGIS software.

**Results:**

Thirty-five studies were selected, highlighting the significant consequences of COVID-19 on TB and DR-TB treatment progress. Four main thematic areas were identified: Clinical and epidemiological aspects of the interaction between COVID-19 and DR-TB; Management of physical resources and the team; Challenges and circumstances; Perspectives and possibilities.

**Conclusions:**

This study revealed that the COVID-19 pandemic significantly negatively impacted the control of long-standing diseases like TB, particularly in the context of morbidity and mortality related to DR-TB.

## RESUMO

**Objetivo:**

Avaliar o impacto da COVID-19 na morbimortalidade associada à tuberculose resistente a medicamentos (DR-TB).

**Métodos:**

Realizou-se uma revisão abrangente de artigos publicados em bases de dados internacionais a partir de dezembro de 2019. As evidências foram apresentadas de maneira narrativa, com o suporte de tabelas, diagramas e um mapa elaborado no software ArcGIS.

**Resultados:**

Foram selecionados 35 estudos que destacaram as consequências significativas da COVID-19 nos avanços no tratamento da TB e da DR-TB. Quatro áreas temáticas foram identificadas: “Aspectos clínicos e epidemiológicos da interação entre COVID-19 e DR-TB”, “Gestão de recursos físicos e da equipe”, “Desafios e circunstâncias” e “Perspectivas e potencialidades”.

**Conclusões:**

Este estudo evidenciou que a pandemia de COVID-19 teve um impacto negativo significativo na progressão do controle de uma doença ancestral como a TB, especialmente no contexto da morbimortalidade por DR-TB.

## RESUMEN

**Objetivo:**

Evaluar el impacto de COVID-19 en la morbilidad y mortalidad asociada con la tuberculosis resistente a medicamentos (DR-TB).

**Métodos:**

Se realizó una revisión integral de artículos publicados en bases de datos internacionales desde diciembre de 2019. Los hallazgos se presentaron de forma narrativa, complementados con tablas, diagramas y un mapa creado con el software ArcGIS.

**Resultados:**

Se seleccionaron 35 estudios que destacaron las consecuencias significativas de COVID-19 en el progreso del tratamiento de la TB y la DR-TB. Se identificaron cuatro áreas temáticas principales: “Aspectos clínicos y epidemiológicos de la interacción entre COVID-19 y DR-TB”, “Gestión de recursos físicos y del equipo”, “Desafíos y circunstancias” y “Perspectivas y posibilidades”.

**Conclusiones:**

Este estudio reveló que la pandemia de COVID-19 tuvo un impacto negativo significativo en el progreso del control de enfermedades antiguas como la TB, especialmente en el contexto de la morbilidad y mortalidad relacionada con la DR-TB.

## INTRODUCTION

In the current context of the COVID-19 pandemic, many challenges have arisen and still persist in global public health, primarily due to the scale of this situation. On March 11, 2020, the World Health Organization declared the SARS-CoV-2 virus a pandemic due to its high contagion rate^(1)^.

One of the major issues that emerged alongside the pandemic is the association between COVID-19 and other diseases that were already a public health concern, such as drug-resistant tuberculosis (DR-TB). DR-TB is the drug resistance of Mycobacterium tuberculosis to drugs used in TB treatment, threatening global disease control efforts. DR-TB can be classified according to the type of drug it is resistant to, with tuberculosis resistant to rifampicin (TB-RR) having resistance to rifampicin; multidrug-resistant tuberculosis (MDR-TB) displaying simultaneous resistance to rifampicin and isoniazid; and extensively drug-resistant tuberculosis (XDR-TB) showing additional resistance to a fluoroquinolone and a secondline injectable drug^(2, 3, 4, 5)^.

TB is a disease with signs and symptoms very similar to COVID-19, making a differential diagnosis difficult. Both diseases may also coexist simultaneously. A study conducted in South Africa suggests that the outcomes of SARS-CoV-2 infection are worse for patients co-infected with TB and MDR-TB, a combination that can be fatal due to the severity of these conditions. Another issue is that TB is exacerbated by its relationship with socioeconomic conditions and social vulnerability, which the COVID-19 pandemic has impacted. Moreover, the pandemic increased the existing healthcare system burden, directly interfering with TB prevention, diagnosis, and treatment^(4, 5, 6, 7, 8, 9)^.

In the pandemic context, evidence has shown that the pandemic significantly influenced the reduction of patient adherence to TB treatment, both due to social isolation and the fear of exposure and attending public places, thereby reducing access to medications and proper treatment^(5, 10, 11)^.

In addition to the access challenges, the pandemic has had a negative impact on the economy, resulting in unemployment and worsening poverty. This has led to worse health inequality indicators and social determinants, which may cause an increase in TB cases and the spread of MDR-TB worldwide. This creates a perfect condition for drug resistance to emerge, resulting in pulmonary comorbidities that lead to poor outcomes in the treatment of these patients when they contract COVID-19 ^(7, 8, 9)^.

Estimates suggest that over the next 35 years, DR-TB will kill approximately 75 million people, at a cost of $16.7 trillion to the global economy. According to a report from the Stop TB Partnership, published in collaboration with the Imperial College, Avenir Health, Johns Hopkins University, and the United States Agency for International Development, efforts to combat tuberculosis may regress even further during the COVID-19 pandemic, with an estimated increase of 6.3 million new TB cases and an increase of 1.4 million TB-related deaths worldwide between 2020 and 2025. This is due to the lack of resources and forced confinement in TB-endemic areas during the pandemic^(4, 12, 13, 14)^.

Therefore, research aimed at studying areas with vulnerable populations and these diseases holds great scientific value, primarily in assisting planning efforts to combat and control these diseases and their impacts on health.

## OBJECTIVE

To assess the impact of COVID-19 on morbidity and mortality associated with drug-resistant tuberculosis (DR-TB).

## METHODS

### Research Design

This is a knowledge synthesis research following the methodological approach of the Joanna Briggs Institute (JBI), using a scoping review protocol. Scoping reviews are useful for examining emerging evidence on a particular subject^(15)^. They are justified by the need to identify the types of evidence available in a specific field, with the aim of detecting and analyzing knowledge gaps, clarifying key concepts or definitions in the literature, examining how research is conducted on a specific topic or field, and understanding the main characteristics or factors related to a concept^(16)^. Unlike other methodological studies, scoping reviews do not aim to assess the methodological quality of the selected studies or identify the best scientific evidence. Instead, they map the existing scientific evidence on a particular subject or event^(17)^.

### Procedure Protocol Adopted

Considering the context before the pandemic and the high vulnerability of specific populations, this study aimed to map scientific evidence that could answer the following guiding question: What is the extent of the impact of COVID-19 in the context of morbidity and mortality associated with DR-TB? This question was developed using the Population (P), Concept (C), Context (C) mnemonic, also known as PCC. Thus, the study was defined according to the Descriptors in Health Sciences/Medical Subject Headings (DeCS/MeSH) for each item, as follows: P - Tuberculosis OR Mycobacterium Tuberculosis; C - Extensively Drug-Resistant Tuberculosis OR Tuberculosis, Multidrug-Resistant OR Mycobacterium Tuberculosis, Susceptibility to Infection by Drug Resistance OR Drug Resistance, Bacterial OR Drug Resistance, Microbial OR Drug Resistance, Multiple; C - COVID-19 OR 2019-nCoV Infection OR Coronavirus Disease 2019 OR COVID-19 Pandemic OR 2019-nCoV Disease OR 2019 Novel Coronavirus Disease OR 2019 Novel Coronavirus Infection OR Coronavirus Disease-19

After delineating the above aspects, the research protocol was registered on the Open Science Framework (OSF) platform with an openly accessible identifier through the link: https://doi.org/10.17605/OSF.IO/3P8MF, as recommended by JBI (2020).

The following databases were considered for the search: National Library of Medicine’s (NLM/PubMed), SciVerse Scopus (Elsevier), Web of Science (WoS), Virtual Health Library (BVS)

The Boolean operators AND and OR were used to structure the search equation. Chart 1 depicts the search equation used for each database and their specificities.

**Chart 1:** Search strategy according to the descriptors and Boolean operators used in each database

Data base	Search
National Library of Medicine’s (NLM/PubMed)	((tuberculosis OR Mycobacterium tuberculosis) AND (Extensively Drug- Resistant Tuberculosis OR Tuberculosis, Multidrug-Resistant OR Mycobacterium tuberculosis, susceptibility to infection by Drug Resistance OR Drug Resistance, Bacterial OR Drug Resistance, Microbial OR Drug Resistance, Multiple)) AND (COVID-19 OR 2019-nCoV infection OR coronavirus disease 2019 OR COVID-19 pandemic OR 2019-nCoV disease OR 2019 novel coronavirus disease OR 2019 novel coronavirus infection OR coronavirus disease-19)
SciVerse Scopus (Elsevier)	( TITLE-ABS-KEY ( tuberculosis ) OR TITLEABS-KEY (mycobacterium AND tuberculosis ) AND TITLE-ABS-KEY ( extensively AND drug-resistant AND tuberculosis ) OR TITLE-ABS-KEY ( extensively AND drug-resistant AND tuberculosis ) OR TITLE-ABS- KEY ( mycobacterium AND tuberculosis, AND susceptibility AND to AND infection AND by AND drug AND resistance ) OR TITLE-ABS- KEY ( drug AND resistance, AND bacterial ) OR TITLE-ABS-KEY ( drug AND resistance, AND microbial ) OR TITLE-ABS-KEY ( drug AND resistance, AND multiple ) AND TITLE-ABS-KEY ( covid-19 ) OR TITLEABS-KEY ( corvidae ) OR TITLE-ABS-KEY ( 2019-ncov AND infection) OR TITLE-ABS-KEY ( coronavirus AND disease 2019 ) OR TITLE-ABS-KEY ( covid-19 AND pandemic ) OR TITLE-ABS-KEY (2019-ncov AND disease) OR TITLEABS-KEY ( 2019 novel AND coronavirus AND disease ) OR TITLE-ABS-KEY ( 2019 novel AND coronavirus AND infection ) OR TITLE-ABS-KEY ( coronavirus AND disease-19 ) )
Web of Science (WoS)	((ALL=(tuberculosis OR Mycobacterium tuberculosis)) AND ALL=(Extensively Drug-Resistant Tuberculosis OR Tuberculosis, Multidrug-Resistant OR Mycobacterium tuberculosis, susceptibility to infection by Drug Resistance OR Drug Resistance, Bacterial OR Drug Resistance, Microbial OR Drug Resistance, Multiple)) AND ALL=(COVID-19 OR 2019-nCoV infection OR coronavirus disease 2019 OR COVID-19 pandemic OR 2019-nCoV disease OR 2019 novel coronavirus disease OR 2019 novel coronavirus infection OR coronavirus disease-19
Virtual Health Library	((tuberculosis) OR (mycobacterium tuberculosis) AND (extensively drug- resistant tuberculosis) OR (tuberculosis, multidrug-resistant) OR (mycobacterium tuberculosis, susceptibility TO infection by drug resistance) OR (drug resistance, bacterial) OR (drug resistance, microbial) OR (drug resistance, multiple) AND (covid-19) OR (covid19) OR (2019- ncov infection) OR (coronavirus disease 2019) OR (covid-19 pandemic) OR (2019-ncov disease) OR (2019 novel coronavirus disease) OR (2019 novel coronavirus infection) OR (coronavirus disease-19))

The search was conducted between March 8 and March 18, 2022, using the criterion of publications from December 2019, which is when the COVID-19 virus was identified. As a language criterion, works in English, Portuguese, and Spanish were chosen. The selection of studies occurred after a thorough review of titles and abstracts. Articles that, in their titles and abstracts, addressed the guiding question, were selected for full-text reading. Subsequently, content extraction was performed using a structured tool created by the authors. The evidence resulting from this process was presented in a diagrammatic manner, following the 2020 Preferred Reporting Items for Systematic Reviews and Meta-Analyses (PRISMA) Flow Diagram recommendations, with tables showing absolute and relative frequency, as well as the representation of the research origins through choropleth maps created in ArcGIS software version 10.6^(18)^. After a comprehensive review of the selected works, the studies were grouped into thematic areas based on the affinity of the content covered.

## RESULTS

**Figure 1:**
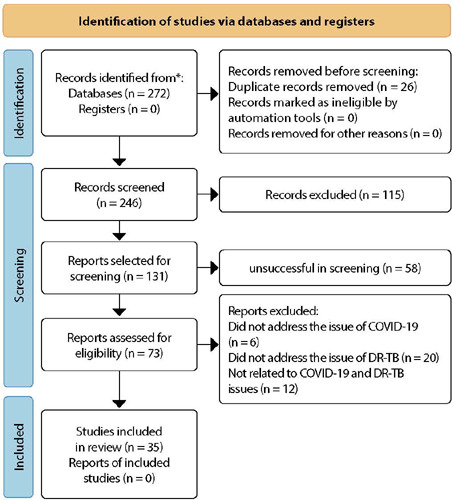
Diagram of the Preferred Reporting Items for Systematic Reviews and Meta-Analyses (PRISMA) 2020^(18)^ with a detailed overview of the research stages conducted, Brazil, 2023

Figure 1 depicts the diagram of the steps taken in the process of selecting articles relevant to our research.

Health Sciences Descriptors/Medical Subject Headings (DeCS/MeSH) for each item were as follows: P - Tuberculosis OR Mycobacterium Tuberculosis; C - Extensively Drug-Resistant Tuberculosis OR Tuberculosis, Multidrug-Resistant OR Mycobacterium Tuberculosis, Susceptibility to Infection by Drug Resistance OR Drug Resistance, Bacterial OR Drug Resistance, Microbial OR Drug Resistance, Multiple; C - COVID-19 OR 2019-nCoV Infection OR Coronavirus Disease 2019 OR COVID-19 Pandemic OR 2019-nCoV Disease OR 2019 Novel Coronavirus Disease OR 2019 Novel Coronavirus Infection OR Coronavirus Disease-19.

From the selected databases, the following studies were relevant to this research: 11 studies in the SCOPUS database, 23 studies in PubMed, 1 study in the Web of Science, and 0 studies in the Virtual Health Library (BVS).

**Figure 2:**
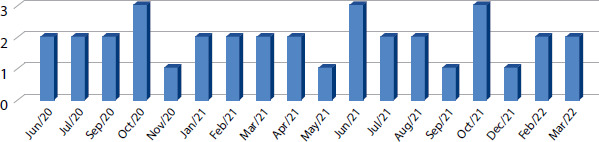
Quantitative description of the selected works by month and year of publication, Brazil, 2023

Regarding the publication period of the studies, relevant studies were published from June 2020 to March 2022, with the months of October 2020, June 2021, and October 2021 being the periods with the highest publication, representing n=3; 8.57% of the studies each month. In contrast, November 2020 and the months of May, September, and December 2021 had the lowest publication rates, with only one (2.86%) article published in each of these months, totaling n=4; 11.42%. The categorization of the periods (month and year) is shown in Figure 2.

Regarding the research design, out of the 35 selected articles, 13 (37.14%) were letters to the editor or comments; 11 (31.42%) represented literature review designs, and 2 (5.71%) were qualitative studies. Only 3 (8.57%) articles had a case report design for DR-TB and COVID-19; 3 (8.57%) were presented as descriptive observational epidemiological studies; 2 (5.71%) were retrospective cohort studies, and only 1 (2.86%) article was multicenter with an ecological design involving ten different countries. Table 1 outlines the studies according to their designs.

**Table 1:** Description of the selected works according to study type, frequency, and percentage, Brazil, 2023

Study Type	n	%	Studies
Letter to the editor or comment	13	37.14	E1^(19)^, E4^(20)^, E7^(4)^, E9^(21)^, E10^(22)^, E13^(13)^, E14^(23)^, E15^(24)^, E16^(7)^, E17^(25)^, E18^(26)^, E21^(27)^, E25^(28)^
Reviews	11	31.42	E2^(10)^, E3^(29)^, E6^(12)^, E8^(30)^, E11^(6)^, E20^(31)^, E22^(32)^, E24^(33)^, E28^(8)^, E31^(34)^, E34^(35)^
Qualitative	2	5.71	E27^(36)^, E35^(9)^
Case report	3	8.57	E5^(37)^, E26^(38)^, E30^(39)^
Descriptive observational	3	8.57	E19^(40)^, E23^(41)^, E33^(11)^
Retrospective cohort	2	5.71	E12^(42)^, E32^(5)^
Ecological	1	2.86	E29^(46)^
Total	35	100	...

In terms of the countries where the studies were published, they were categorized by continents, as shown in Figure 2. The frequency of countries within each continent is detailed below:

In Asia, the study included the following countries: China (2), India (4), Pakistan (1), Indonesia (1), and Vietnam (1). In Africa, the recorded countries were South Africa (3), Ethiopia (1), and Sierra Leone (1). In Latin America, the study observed the following countries: Haiti (1), Paraguay (1), and Brazil (2).

However, 17 studies did not specify their countries of origin. Out of these, 10 were systematic reviews, which typically compile studies from various countries. Among the remaining 17 studies, 4 were letters to the editor, 2 were descriptive observational studies, and 1 was an ecological study that did not mention its place of origin.

Concerning research funding, only thirteen (37.14%) studies received financial support out of the total. Among these, six (46.15%) were systematic reviews, three (23.07%) were letters to the editor or comments, two (15.38%) were qualitative studies, one (7.70%) was a retrospective cohort study, and one (7.70%) was a multicenter ecological study. The remaining studies (n=22, 62.86%) either did not secure funding or did not disclose this information.

As described in the methods, following a comprehensive review of the selected works, the studies were categorized into thematic areas based on the affinity of their content. This categorization makes it easier for readers to grasp the different dimensions of this research. It was anticipated that studies might address multiple thematic areas.

The first common thematic area identified in the selected studies was titled “Clinical and Epidemiological Aspects of the COVID-19 and DR-TB Interaction.” This theme was mentioned in 19 (54.28%) of the studies. It was followed by “Management of Physical and Team Resources” with 12 (34.28%) studies, “Challenges and Circumstances” with 8 (22.85%) studies, and finally, “Advancements and Potentials” with 10 (28.57%) studies.

**Figure 3:**
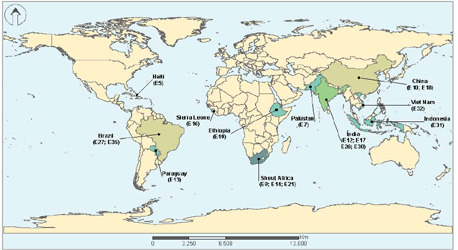
Distribution of selected works according to the country where the research was conducted, Brazil, 2023

## DISCUSSION

In the pursuit of answers to the research question, “What is the magnitude of COVID-19 in the context of morbidity and mortality related to DR-TB?” the scientific works identified were unanimous in illustrating the impact of COVID-19 on exacerbating one of the crucial aspects for TB control, DR-TB. The descriptive elements of the study are linked to the period when the coronavirus was declared a pandemic^(1)^. This context indicates that the primary focus of the studies was on the mechanisms of transmission, treatment, and control of COVID-19. As more information was acquired, new questions arose concerning the potential direct and indirect effects of the disease in various health domains, including concerns about the future of antimicrobial resistance following significant changes in the healthcare system^(28)^.

According to Preez et al.^(35)^, the connection between COVID-19 and other diseases and events is still a relatively unexplored topic in scientific literature. This may be attributed to the limited number of publications related to the research question (only 35 out of 246 identified works).

Despite the syndemic challenges posed by the pandemic, among the findings, the earliest mentions connecting COVID-19 events and DR-TB were published in June 2020, originating from London^(20)^ and Sierra Leone^(7)^, respectively, in the form of comments and editorials. It’s also noteworthy that the publication of studies can be influenced by the eligible timeframe of the works, as the results indicate a limited number of epidemiological studies, and none with a clinical design.

Regarding the regions where the studies were conducted, there is a clear association with social and health determinants, as most of the research originates from areas facing significant challenges in TB control. To illustrate, among the ten countries representing 75% of the global TB burden^(2)^, six of them are featured in the selected works: China, India, Pakistan, Indonesia, Vietnam, and South Africa. Three studies were conducted in countries among the 30 high TB burden countries, specifically Sierra Leone, Brazil, and Ethiopia. Additionally, two more studies took place in Haiti and Paraguay. While these two countries are not on the list of high TB burden countries, they are still considered developing countries. Haiti has an impoverished population living on less than $1 per day, a high TB incidence (188/100,000), and 15% of diagnosed TB patients are co-infected with HIV^(22)^. Paraguay, on the other hand, grapples with income distribution disparities, a significant poverty problem, and a reported TB incidence rate of up to 42/100,000 cases per inhabitant^(44)^.

Studies^(2, 4)^ indicate that, even after the emergence of COVID-19, TB continues to be one of the leading causes of death from a single infectious agent. In 2021, approximately 10.6 million new TB cases were diagnosed, with 1.6 million deaths, representing a 4.5% increase compared to 2020.

Upon reviewing the studies included in this article, it becomes evident that both the severity of COVID-19 and TB has increased. The COVID-19 pandemic significantly curtailed access to TB diagnosis and treatment services, resulting in increased TB-related deaths and a significant setback in the global fight against TB^(9, 11)^.

While there are no studies that provide a comprehensive understanding of the consequences of the association between COVID-19 and TB, it’s essential to consider that both Mycobacterium tuberculosis and SARS-CoV-2 primarily target the lungs, potentially leading to complications such as hemoptysis, pneumothorax, mediastinal emphysema, and fungal infections^(9, 26)^. Furthermore, patients with lung sequelae after TB may experience exacerbated conditions when contracting COVID-19, particularly in cases of multidrug-resistant TB (MDR-TB)^(8, 27, 39)^.

It’s worth highlighting that, despite its viral origin, COVID-19 often leads to the widespread use of antimicrobials, disregarding WHO recommendations. Authors have reported that about 50% of COVID-19 deaths analyzed in their studies had secondary bacterial infections^(45)^. This has worsened the problem of Antimicrobial Resistance (AMR) and, as a result, DR-TB, in two ways: through increased use and decreased production of antimicrobials, causing shortages and necessitating adaptations in the use of antimicrobial agents. This includes broad-spectrum use when the recommended option is unavailable or suboptimal dosing due to a lack of access to complete treatment. Additionally, there have been concerns about questionable quality and counterfeit medicines^(20, 29, 31)^.

Given the diversity of severe consequences evident in the eligible works, it was observed that publications followed patterns of disease associations. This led to the creation of four relevant thematic areas for discussion:

### Clinical and Epidemiological Aspects of the COVID-19 and DR-TB Interaction

It is widely recognized that approximately 18% of individuals who initiate TB treatment develop resistance to key medications. Therefore, an issue that was already problematic before the pandemic has become even more critical during it^(29)^. Studies indicate that patients with MDR-TB are more susceptible to COVID-19 and experience worse outcomes due to their already compromised health conditions. This is compounded by difficulties in accessing healthcare services, discontinuation of directly observed treatment (DOT), shortages of healthcare professionals, reduced appointments to minimize exposure to SARS-CoV-2, and diminished notifications of new cases. Moreover, the stigma associated with respiratory symptoms, already faced by TB patients, has intensified due to the additional threat of COVID-19^(4, 24, 30)^. As a result, delays in diagnosis and all the mentioned challenges have increased the likelihood of incomplete therapies^(4, 6, 7, 20, 34, 41, 42)^.

The TB case notification rate dropped by more than 23% in high TB burden countries, resulting in 1 million undetected cases. Globally, the number of diagnosed and reported TB cases decreased by 22%, and the number of people in MDR-TB treatment decreased by 15%. Predictions suggest that TB deaths may increase by 20% over the next 5 years, translating to an additional 4,000 TB-related deaths, while TB incidence may rise by 3% to 9%^(11, 33, 41)^.

Another significant concern is the resurgence of childhood TB, contributing to the ongoing transmission chain. Authors^(7, 23)^ point out that children with TB are at risk of being not only neglected but also forgotten. In Indonesia, the second country with the highest incidence of childhood TB, there was a 116% increase in the number of children who died during TB treatment in 2020 due to delayed access to healthcare^(35)^. A study conducted in Paraguay by Coronel, Aguirre, and Pérez (2020)^(13)^ compared the first half of 2019 with the same period in 2020 and observed a reduction of over 80% in notifications of patients with respiratory symptoms and a 42.4% decrease in the confirmation of TB diagnoses. These facts may lead to long-term issues in the response to TB since a lack of timely diagnosis results in treatment failures and an increased number of DR-TB cases^(13, 34, 36)^.

A similar situation was reported in Vietnam by Hasan et al. (2022)^(5)^, who observed a decline in notifications and TB smears and cultures, suggesting a potential delay in diagnosing TB and DR-TB cases. Late case detection can lead to more advanced TB cases even before diagnosis^(5)^. Arega et al. (2022)^(40)^ warn about the consequences of increased rates of latent infection, ensuring the continuation of this dramatic situation and the potential rise in fatality.

According to Tamuzi et al. (2020)^(30)^, the exaggerated reactivation of latent TB can occur due to the excessive use of corticosteroids in cases of SARS-CoV, due to the transient suppression of cellular immunity, which makes patients more susceptible. In the context of increased fatality, clinical factors demand more complex healthcare services, such as hospitalizations. In a preliminary study^(6)^ involving approximately 40 countries, it was observed that out of the 381 patients under surveillance, 222 (58%) were hospitalized due to TB. Of these hospitalizations, 82 (37%) involved TB and COVID-19 coinfected patients, with approximately 66 (80%) being DR-TB patients. These findings align with global antimicrobial resistance concerns, as one-third of antimicrobial resistance-related deaths worldwide are linked to TB^(33)^.

Gao et al. (2021)^(46)^ and Wingfield et al. (2021)^(33)^ emphasize that while TB patients are not at an increased risk of acquiring COVID-19, coinfection makes COVID-19 twice as severe. According to Tamuzi et al. (2020)^(30)^, the interaction between fibrosis or extensive pulmonary pathology due to TB and COVID-19 may reduce drug penetration at pulmonary sites, representing a significant risk for DR-TB, MDR-TB, or extensively drug-resistant TB (XDR-TB).

### Management of Physical and Team Resources

It is evident that, as in any war, management is always necessary, and in the fight against COVID-19, it was no different. Out of the 35 eligible works, 12 included sections related to the management of physical and team resources. The COVID-19 pandemic reshaped the world^(30)^ in various ways. Low- and middle-income countries, which already had fragile and overwhelmed healthcare systems, were even more affected, depleting the availability of resources for the prevention and management of other infectious diseases^(20, 46)^.

Among the insights related to this area, the organization of hospitals and health centers for COVID-19 care/isolation and the reallocation of professionals were the most frequent^(9, 25, 27, 28, 33, 36, 38, 40, 41, 43)^. It is important to note that among the different healthcare settings, such as primary care, emergency departments, hospitals, and others, nursing professionals are the ones who maintain the first contact with the clientele^(47)^. Since the early discussions of the Sustainable Development Goals (SDGs), the need to invest in education, employment, and leadership in the nursing profession has been emphasized to increase the availability of universal healthcare services^(1)^. The findings of this research support the importance of this need and also highlight the need to address emergency situations involving the prevention and combat of outbreaks, endemic diseases, epidemics, and future pandemics.

Less frequently, but still relevant, one study^(20)^ reported the shortage of supplies for the prevention and management of other infectious diseases. Furthermore, more related to the issue of DR-TB, the use of TB diagnostic services for COVID-19 was highlighted, such as GeneXpert machines (Cepheid, Sunnyvale, California, United States), the redefinition of research, development, and administration priorities for Bacillus Calmette-Guérin vaccination, and the disruption in the availability and access to medicines and supplies^(28)^.

### Diligence and Scenarios

Discussing the diligence and scenarios following the occurrence of cases can generate a significant dichotomy. However, COVID-19 cases continued to increase during the preparation of this article, and some points can still be incorporated based on reports and observations regarding adaptations due to the pandemic. In this context, there were various articles with insights that ranged from clinical behaviors to various needs.

According to Coronel, Aguirre, and Pérez (2020)^(13)^, the advent of COVID-19 represents one of the great opportunities to finally learn about a new disease without forgetting a much older one. To achieve this, it is necessary to take real and concrete actions for the effective control of TB and, consequently, DR-TB. Knight et al. (2021)^(29)^ emphasize the importance of leveraging the momentum that the pandemic has given to make science even more open, with results shared among scientific groups and institutes.

In this regard, there were also findings highlighting the primary need to provide TB preventive therapy for all clinical forms, including contacts, as an emergency measure to combat the devastating impact of the pandemic on this ancient disease^(35)^. However, Knight et al. (2021)^(29)^ warn that the situation requires strengthening clinical treatment guidelines to limit unnecessary exposure to antimicrobials, both in the case of COVID-19 and TB since global drug-resistant TB still poses a threat to public health^(11)^.

Parums (2021)^(28)^ reinforces that identifying DR-TB requires careful monitoring and resources for performing molecular tests to detect resistance, which are not always easily accessible to everyone. Additionally, the imminent development of drugs and the use of shorter treatment regimens for DR-TB patients have become even more evident.

Among clinical diligences, Liu et al. (2020)^(26)^ emphasize the importance of not administering corticosteroids when there is a COVID-19/TB coinfection, due to the possibility of both causing immunosuppression. Although this recommendation still needs broader confirmation, it also warrants a broader understanding, as highlighted in the first sub-item of this article’s discussion.

Another insightful finding is the recommendation for the development of specialized screening guidelines and protocols for TB/DR-TB/COVID-19^(37)^, including requesting TB laboratory tests in cases of COVID-19^(40)^ and lower respiratory tract samples (such as sputum, bronchoalveolar lavage, and tracheal aspirates) in patients with suspected COVID-19/TB, as well as increasing the availability of the GeneXpert MTB/RIF Rapid Molecular Test, which produces results in about two hours, while COVID-19 RT-PCR can take up to 24 hours^(22, 30)^.

### Advancements and Potential Opportunities

As previously mentioned, the scientific momentum resulting from the pandemic should be seized^(29)^, and various advancements have been identified amid numerous challenges. These include the reinforcement of Web-DOT^(36)^ and the expansion of digital adherence technologies (DATs)^(10, 40)^ whenever feasible. Additionally, discussions related to the need for genetic sequencing have gained increased popularity and dissemination^(^12,^, 48)^, alongside progress in the use of drugs such as bedaquiline and delamanid for DR-TB cases^(19, 20, 21, 24)^, and hypotheses regarding the potential use of the Bacillus Calmette-Guérin vaccine as support for COVID-19 control^(41, 42)^.

### Study Limitations

Despite the substantial contribution this study has made to understanding the interaction between COVID-19 and drug-resistant tuberculosis (DR-TB), it has some limitations. One of these limitations is the reliance on open-access sources, which may have excluded relevant research not available in this format. Furthermore, the analysis was based on data available up to the cutoff date, and new research and discoveries may have emerged since then, impacting the comprehensiveness and currency of the information. It is also worth noting that the heterogeneity in the quality and scope of the included studies may introduce biases in evidence synthesis. Therefore, while this study provides a comprehensive overview of the subject, these limitations underscore the ongoing need for further research and review as more data becomes available and our understanding of the interaction between COVID-19 and DR-TB continues to evolve.

### Contributions to the Nursing Field

Given that care is the epistemological focus of the nursing profession, nursing plays a strategic role in care management^(49)^. To fulfill this role, nursing professionals must integrate care and management actions, prioritizing care based on human needs^(50)^. In this context, knowledge synthesis products like this work serve as fundamental tools to empower nursing professionals to practice based on comprehensive evidence and grounded in cooperation, coordination, and interdisciplinarity.

## CONCLUSIONS

Our study has revealed compelling evidence of the impact of the COVID-19 pandemic on DR-TB morbidity and mortality. This has led to a significant setback in achieving the desired progress in the control of an age-old disease like tuberculosis, where DR-TB is one of the most pressing challenges. In the extensive landscape of identified challenges, opportunities have also emerged with the potential to enrich our understanding of the clinical and epidemiological aspects of the interaction between these two diseases. Additionally, opportunities for improving the management of physical and team resources to optimize healthcare delivery are evident. As a result, the findings indicate advancements and potential opportunities identified thus far, which can effectively reshape the delivery of healthcare in the context of tuberculosis, drug resistance, and even COVID-19.
